# When-to-Loop: Enhanced Loop Closure for LiDAR SLAM in Urban Environments Based on SCAN CONTEXT

**DOI:** 10.3390/mi15101212

**Published:** 2024-09-29

**Authors:** Xu Xu, Lianwu Guan, Jianhui Zeng, Yunlong Sun, Yanbin Gao, Qiang Li

**Affiliations:** 1 College of Intelligent Systems Science and Engineering, Harbin Engineering University, Harbin 150001, China; xuxu66@hrbeu.edu.cn (X.X.); gaoyanbin@hrbeu.edu.cn (Y.G.); 2 China North Vehicle Research Institute, Beijing 100072, China; sunyunlong0124@sina.cn; 3 Chongqing Huayu Electric Group Co., Ltd., Chongqing 400015, China; 13996270865@163.com

**Keywords:** MEMS, LiDAR odometry and mapping, loop closure enhancement, urban environments

## Abstract

Global Navigation Satellite Systems (GNSSs) frequently encounter challenges in providing reliable navigation and positioning within urban canyons due to signal obstruction. Micro-Electro-Mechanical System (MEMS) Inertial Measurement Units (IMUs) offers an alternative for autonomous navigation, but they are susceptible to accumulating errors. To mitigate these influences, a LiDAR-based Simultaneous Localization and Mapping (SLAM) system is often employed. However, these systems face challenges in drift and error accumulation over time. This paper presents a novel approach to loop closure detection within LiDAR-based SLAM, focusing on the identification of previously visited locations to correct time-accumulated errors. Specifically, the proposed method leverages the vehicular drivable area and IMU trajectory to identify significant environmental changes in keyframe selection. This approach differs from conventional methods that only rely on distance or time intervals. Furthermore, the proposed method extends the SCAN CONTEXT algorithm. This technique incorporates the overall distribution of point clouds within a region rather than solely relying on maximum height to establish more robust loop closure constraints. Finally, the effectiveness of the proposed method is validated through experiments conducted on the KITTI dataset with an enhanced accuracy of 6%, and the local scenarios exhibit a remarkable improvement in accuracy of 17%, demonstrating improved robustness in loop closure detection for LiDAR-based SLAM.

## 1. Introduction

Accurate and reliable navigation in complex urban environments is crucial for the advancement of autonomous systems. However, urban canyons, which are characterized by towering structures and dense infrastructure, pose significant challenges to traditional Global Navigation Satellite System (GNSS)-based positioning [1]. These environments frequently disrupt GNSS signals, leading to unreliable and inaccurate positioning information and thereby necessitating alternative solutions for robust urban navigation. To address these challenges, Micro-Electro-Mechanical System (MEMS) Inertial Measurement Units (IMUs), which are compact and cost-effective inertial sensors, have emerged as essential components for vehicular navigation in these demanding urban scenarios [1,2]. MEMS-IMUs, typically comprising accelerometers and gyroscopes, provide high-frequency motion data independent of external environmental factors, enabling the continuous tracking of vehicular movement. This inherent independence from external influences makes MEMS-IMUs particularly valuable in GNSS-denied environments.

**Figure 1:**
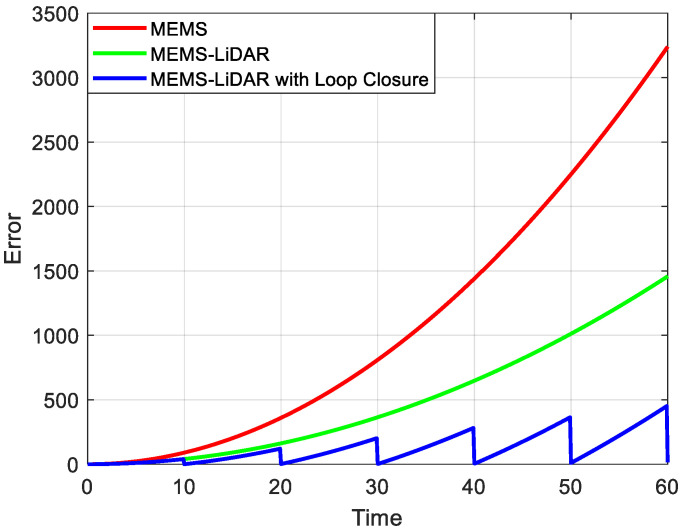
Illustration of Navigation Error Divergence over Time.

However, MEMS-IMUs are not without limitations. The inherent drift and bias within MEMS-IMU measurements, stemming from sensor imperfections and environmental variations, result in significant time-accumulated deviations in position and orientation estimates [3]. This cumulative error poses a substantial challenge for long-term navigation accuracy, as illustrated by the red line in Figure 1. To mitigate this challenge, Simultaneous Localization and Mapping (SLAM) technology has been introduced, incorporating exteroceptive sensors like cameras and Light Detection and Ranging (LiDAR) [4]. LiDAR has gained prominence due to its ability to provide accurate depth information, even in adverse weather conditions and low-light scenarios. This robustness makes LiDAR a more robust and versatile solution for augmenting IMU data and achieving accurate and reliable localization and mapping in challenging urban environments [5,6].

The advent of LiDAR-based SLAM has spurred extensive research into efficient and accurate point cloud processing algorithms. Early approaches relied on traditional methods like Normal Distributions Transform (NDT) [7] and Iterative Closest Point (ICP) [8]. While NDT has proven effective in 2D LiDAR SLAM [9], its direct application to 3D data faces computational challenges due to the increased complexity and size of 3D point clouds. Variations like SEO-NDT [10] and KD2D-NDT [11] have attempted to address these computational bottlenecks, but they often introduce trade-offs in accuracy or robustness. Similarly, ICP-based methods that aim for optimal alignment between point clouds have undergone significant refinements to handle the complexities of 3D data and meet the real-time performance demands of LiDAR SLAM. Notable advancements include the Generalized ICP (G-ICP) algorithm [12], which incorporates surface normal information for improved alignment, and the point-to-plane ICP variant, which is known for its computational efficiency.

A significant breakthrough in LiDAR SLAM came with the development of the LiDAR Odometry and Mapping (LOAM) algorithm [13]. LOAM strategically focuses on processing feature-rich points within the point cloud, specifically edge points and planar points, to enhance both speed and accuracy. This approach allows for efficient extraction of geometric features, leading to improved odometry and mapping performance. However, LOAM also displays the limitations of relying solely on LiDAR for SLAM. While LiDAR provides accurate but relatively low-frequency measurements (up to 10 Hz), its performance can be hampered by motion distortion and requires complementary data, such as from a MEMS-IMU, for optimal results. This limitation reveals the potential synergy between LiDAR and MEMS sensors in SLAM applications, where the strengths of each sensor can compensate for the weaknesses of the other. Although LOAM can maintain accuracy within a limited area, as illustrated by the green line in Figure 1, long-term drift still accumulates over time, indicating the need for further optimization, particularly in handling large-scale environments.

Loop closure detection plays a crucial role in mitigating the inevitable drift accumulation inherent in SLAM systems [14]. By enabling sensors like cameras and LiDAR to recognize previously visited locations, loop closure allows for the correction of accumulated errors and the creation of globally consistent maps. As demonstrated by the blue line in Figure 1, successful loop closure can significantly reduce drift, maintaining high accuracy in navigation and positioning over extended periods. This inherent value of loop closure has led to the creation of many subsequent LiDAR SLAM algorithms, particularly those building upon the strong foundation established by LOAM, to incorporate it as a core component for enhancing long-term performance.

One notable example is LeGO-LOAM, a lightweight and ground-optimized variant of LOAM [14], designed for real-time performance required in ground vehicles. LeGO-LOAM reduces computational complexity and improves efficiency by explicitly segmenting and processing ground points separately. It also incorporates loop closure detection by comparing feature sets of consecutive scans to identify previously visited locations. The pursuit of robust loop closure has driven the exploration of alternative methods beyond LOAM and its variants. SCAN CONTEXT [15], a non-histogram-based approach that constructs a global descriptor directly from the sensor’s field of view, has emerged as a promising solution for 3D LiDAR-based SLAM. Its enhanced version, SCAN CONTEXT++ [16], further improves performance by incorporating ring weights and sector key encoding.

A persistent challenge in loop closure detection lies in the growing computational burden associated with the increasing number of keyframes accumulated over time. Traditional approaches that only consider keyframes as potential candidates for loop closure lead to significant computational overhead as the map grows [17]. To address this issue, ASL-SLAM [18] proposes a novel detection strategy based on motion semantics, focusing loop closure detection on specific events, such as when the LiDAR vehicle traverses speed bumps. This approach leverages IMU data to identify these events by analyzing peak values in the acceleration of the IMU’s z-axis. By intelligently narrowing the search space, ASL-SLAM exemplifies a shift in perspective towards strategically determining when to perform loop closure detection. This leads to the central question explored in this paper: how can we intelligently determine when to perform loop closure detection to maximize its effectiveness while minimizing computational overhead in complex and challenging urban environments?

Building upon the advancements in LiDAR SLAM and the crucial role of loop closure in mitigating drift, a persistent challenge lies in the growing computational burden associated with loop closure detection. While algorithms like LeGO-LOAM and SCAN CONTEXT have demonstrated the effectiveness of loop closure, considering all keyframes as potential candidates leads to significant computational overhead, especially in large-scale environments, as highlighted in [17]. To address this challenge and intelligently determine the optimal moments for loop closure detection, this paper makes the following contributions:

1. LiDAR Scan-Based Road Segmentation for Keyframe Selection: This work proposes a novel approach to classify road segments based on the distribution of LiDAR point cloud data. This classification allows for the strategic selection of keyframes, prioritizing those captured in areas with a higher likelihood of loop closure, such as intersections or areas with distinct features. This approach significantly reduces the number of keyframes considered for loop closure detection, thereby mitigating the computational burden.

2. IMU-Based Motion Semantics for Identifying Potential Loop Closure Events: Leveraging the rich information provided by IMU data, this paper introduces a vehicular motion classification method based on motion semantics. By analyzing patterns in IMU readings, such as acceleration and angular rate, the proposed method can identify the specific maneuvers or events that are indicative of potential loop closures. This further refines the selection of keyframes, focusing on moments where loop closure is more likely to occur.

3. Enhanced SCAN CONTEXT with Structural Information for Robust Loop Closure: After identifying potential loop closure moments using the methods outlined in contributions 1 and 2, this paper enhances the SCAN CONTEXT algorithm by incorporating structural information from the LiDAR point cloud. This richer representation of the environment enables more robust and accurate loop closure detection.

## 2. Methods

This Section begins with an explanation of the overall workflow of the proposed algorithm. It includes an overview of the underlying principles governing the relevant hardware components and a detailed description of the data preprocessing methodologies.

To further clarify the algorithmic details, the following Sections will assume the LiDAR point cloud originates from a 16-line LiDAR system. This assumption reflects commonly used systems like Velodyne’s VLP-16 and the LeiShen MS-C16 [19], which are employed in local experiments. These systems, each with a horizontal angular resolution of 0.2° and a vertical resolution of 2°, generate a range image of 1800 by 16 pixels [19]. This translates to a point cloud with 16 projection planes, each containing 1800 points, which is the basis for the subsequent algorithmic explanations.

### 2.1. Algorithm Overview

**Figure 2:**
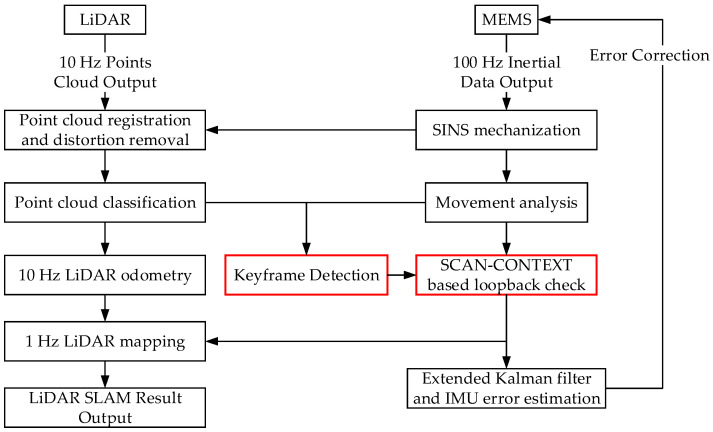
Overview Workflow of Proposed Algorithm.

Figure 2 illustrates the overall workflow of the proposed algorithm. The core components, namely keyframe detection and loop closure extraction, are highlighted in a red rectangular box. The keyframe detection process analyzes data from both LiDAR and MEMS sensors, while the extraction and verification of loop closure information are detailed in Section 2.4. This dual-sensor approach presents a comprehensive understanding of the environment and vehicular motion, leading to the intelligent and efficient selection of keyframes for loop closure detection.

### 2.2. Keyframe Detection Based on LiDAR Point Cloud

**Figure 3:**
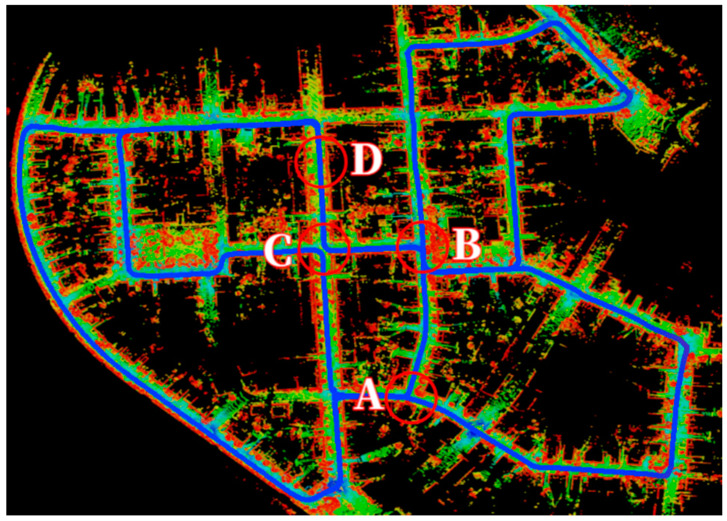
Potential Scenarios for Vehicular Navigation in Urban Environments from KITTI Datasets.

As mentioned in the introduction, one effective way to reduce the computational burden of LiDAR loop closure detection is to minimize the number of loop closure candidate frames. This necessitates a more intelligent approach to determining when keyframes should be stored and compared. Figure 3, sourced from the dataset 00 of the KITTI open-source dataset, depicts four typical scenarios encountered by a vehicle during navigation in a typical urban environment. In scenario A, the vehicle approaches a three-way intersection. In scenario B, the vehicle undergoes a significant change in direction. Scenario C presents a four-way intersection. Finally, scenario D illustrates the vehicle traveling along a straight, unidirectional road.

Analyzing these scenarios, it becomes evident that scenarios A, B, and C occur less frequently and are more representative compared to scenario D. Furthermore, considering the likelihood of revisiting a location, scenarios A and B offer the possibility of passing through the same point from three different directions, while scenario C offers four. In contrast, scenario D only presents two possibilities (forward and backward). This observation indicates that scenarios A, B and C are more prone to loop closure occurrences during regular driving compared to scenario D. Moreover, from the perspective of urban structural features, scenario D exhibits a significantly higher probability of structural repetition compared to the other three scenarios, making it more susceptible to erroneous loop closure detections. From a topological perspective, scenarios A, B, and C can be considered as nodes within the environment, whereas scenario D represents the lines connecting these nodes. The former possess greater uniqueness and representativeness, making it more crucial for loop closure considerations. This observation leads to the central argument of this Section: keyframe extraction for LiDAR loop closure should prioritize scenarios with potential for revisiting, effectively discarding those captured along straight, unidirectional roads.

Therefore, the scenarios encountered by the vehicular navigation can be simplified into two categories: those suitable (multi-path scenarios) and unsuitable (single-path scenarios) for keyframe retention. The most effective way to differentiate between these categories is to classify them directly based on point cloud information, which focuses on the areas where the vehicle can navigate the ground and obstacles. Intuitively, the ground represents the navigable space, while obstacles define the boundaries and constraints of movement.

**Figure 4:**
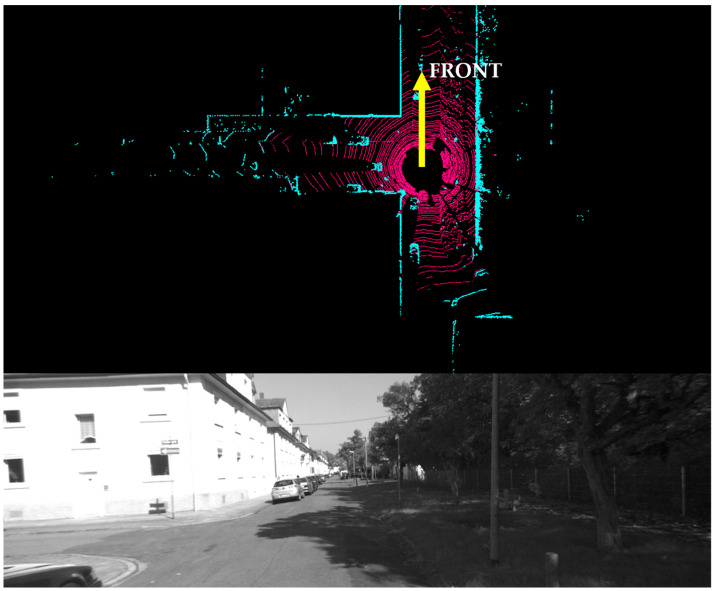
Ground and Obstacle Points in LiDAR Point Cloud and Image from Left Camera.

As illustrated in Figure 4, which shows the LiDAR point cloud and the left camera image corresponding to scenario B of Figure 3, the pink lines represent the navigable ground plane, while the cyan areas depict non-navigable obstacles. The yellow arrow indicates the X-axis of the LiDAR scan, which is forward, and the tail end of the arrow is the center of the LiDAR point cloud. To distinguish between these two conditions, point cloud segmentation is employed. This process involves classifying each point in the point cloud as either belonging to the ground or an obstacle, effectively creating a map of the navigable space. Therefore, the proposed algorithm can identify multi-path scenarios by analyzing the segmented point cloud, which is characterized by branching paths or intersections, and it can prioritize them for keyframe selection.

#### 2.2.1. Ground Points

LeGO-LOAM utilizes a simple and effective approach for extracting ground points, focusing on the detection of ground points by scrutinizing only 8 out of the 16 (half of the line number) lines that are situated below the 0° threshold [11].

**Figure 5:**
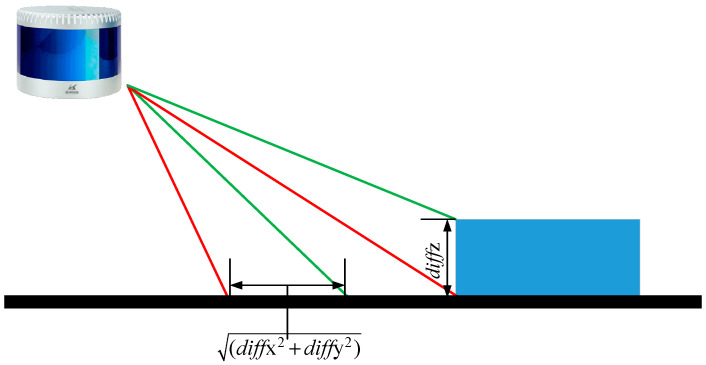
Ground Points Extracted from Point Cloud $\hat{P}$.

In point cloud $\hat{P}$, the point clouds are labeled with rings and scan sequence, and $p_{i,j}\in \hat{P}$, i=1,2,3⋯16;j=1,2,3⋯1800. As shown in Figure 5, to calculate the angle between adjacent points $p_{i,j}$ and $p_{i+1,j}$, this paper assumes that their coordinate differences are denoted as diffx, diffy and diffz. The angle θ can be set as follows:

(1) $$\theta =\tan^{-1}\left(diffz,\;\sqrt{diffx^{2}+diffy^{2}}\right)$$

Once θ<10°, points are marked as candidate ground points. Furthermore, an advanced point cloud sieving process will utilize the RANSAC (Random Sample Consensus) technique to confirm the identification of ground points. This step is critical to avoid the misclassification of non-ground points as ground points, thereby ensuring the accuracy and reliability of the ground detection process. The fitted ground equation is as follows:

(2) Ax+By+Cz+d=0

Then, an image-based segmentation method is applied to the range image to group points into many clusters. Points from the same cluster are assigned to a unique label.

#### 2.2.2. Obstacle Points

Many algorithms, like LeGO-LOAM, typically begin by classifying point cloud data into ground and non-ground points. While this distinction is useful in 3D space, it might be misleading when considering the practical constraints of vehicular navigation. In general, non-ground points are considered obstacles, which is accurate in a 3D context. However, when compressing 3D point cloud data into 2D data for feature extraction and comprehensive analysis, the actual navigable space for the vehicle needs to be considered. Given that only obstacles below a certain height significantly impact the vehicular trajectory and considering the common placement of LiDAR sensors on the vehicular roof, a simplification is proposed and constructed: focusing solely on points within the LiDAR point cloud that fall below a predefined height threshold (LiDAR height + 0.3m). This approach leverages the inherent advantage of the LiDAR sensor’s elevated position to efficiently filter irrelevant data and streamline processing.

**Figure 6:**
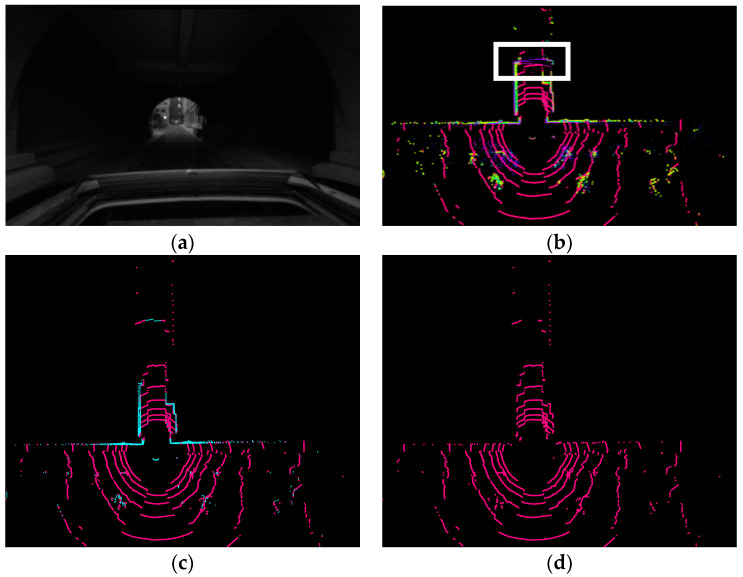
Point Cloud Processing for a Scene in Overhead Occlusion. (**a**) Front View Camera Image. (**b**) Original LiDAR Point Cloud Scan with Overhead Occlusion Highlighted in White. (**c**) Filtered Point Cloud with Distractions like Pedestrians Removed. (**d**) Extracted Ground Points.

Figure 6 illustrates a scenario where a vehicle navigates through a passage between buildings. Figure 6a shows the front view captured by the camera, while Figure 6b depicts the complete point cloud scan from the LiDAR. The vehicle is passing through an area with overhead occlusion, highlighted as the white-marked region in Figure 6b. A simple bird’s-eye view projection and analysis of this data can easily misinterpret the scene as a single or dual-lane straight passage. Figure 6c presents the point cloud after filtering and removing distractions like pedestrians, while Figure 6d shows the extracted ground points using the method described in Section 2.2.1. Figure 6c clearly reveals a three-way intersection, indicating the need to record a keyframe for loop closure detection. However, the scene might be mistakenly categorized as a specific type without loop closure preprocessing.

#### 2.2.3. Keyframe Extraction Based on LiDAR Scanning

**Figure 7:**
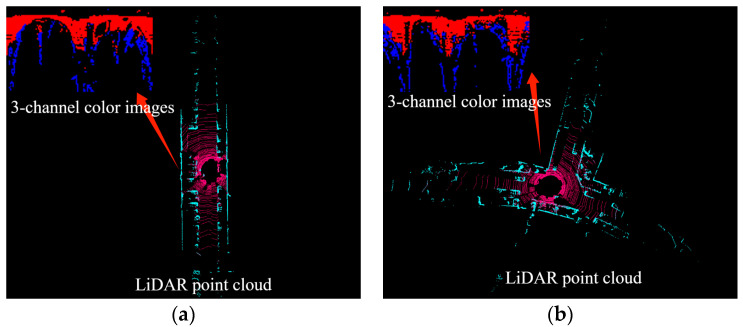

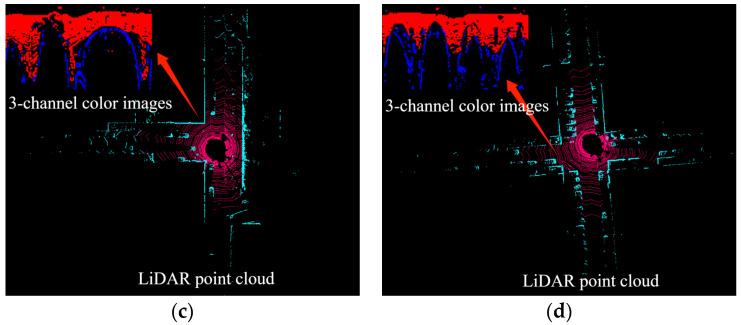
Extracted Ground and Obstacle Point Clouds in Different Scenarios. (**a**) Straight Road. (**b**) T-Junction on a Circular Road. (**c**) T-Junction. (**d**) Four-Way Intersection.

**Figure 8:**
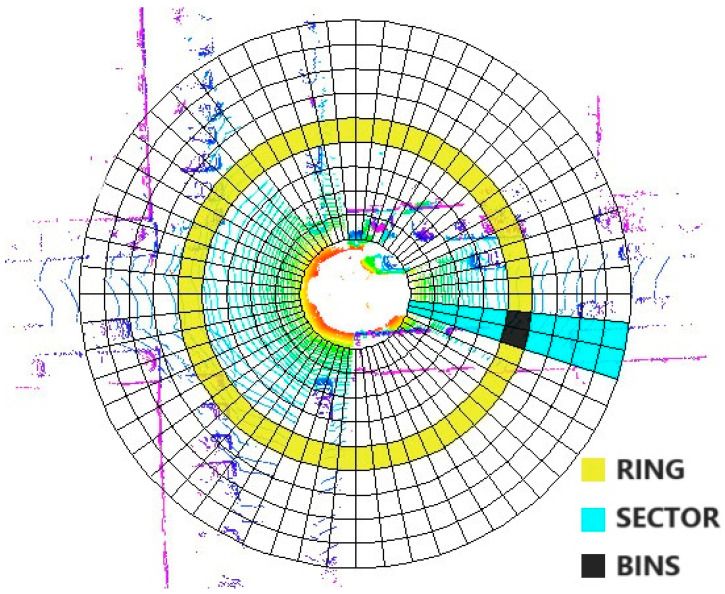
SCAN CONTEXT Bins.

Figure 7 displays the distribution of point clouds in four different scenarios; to further determine the characteristics, it is necessary to process the point cloud with SCAN CONTEXT treatment. Figure 8 shows the bin division along azimuthal and radial directions with the top view of a point cloud from a 3D scan. The ground areas are split in the azimuthal (from 0 to 2π within a LiDAR frame) and radial (from the center to maximum sensing range) directions. They refer to the yellow area as a ring, the cyan area as a sector, and the black-filled area as a bin [15]. 

In this Section, the heights of the extracted point clouds are uniformly set to 0 for compression. Then, follow the convention where each horizontal axis represents 3 degrees and each vertical axis represents 0.5 m. For the bins within, a counting method is used to determine the characteristics: if there are no ground points or obstacles in the bin, it is recorded as 0. When the number of ground points is greater than the obstacle points, it is recorded as 1. If there are fewer obstacle points than ground points, it is recorded as 2. Thereafter, the point cloud within a range of 35 m can be compressed into a 120 × 70 two-dimensional array. To visualize the compressed point cloud data in Figure 7, this paper utilizes a three-color representation of the arrays. Data points marked as ‘0’ are represented by black pixels in the image, red pixels correspond to data points marked as ‘1’, and blue pixels represent data points marked as ‘2’. The resulting visualization is displayed in the top-left Section of the four subfigures in Figure 7.

**Figure 9:**
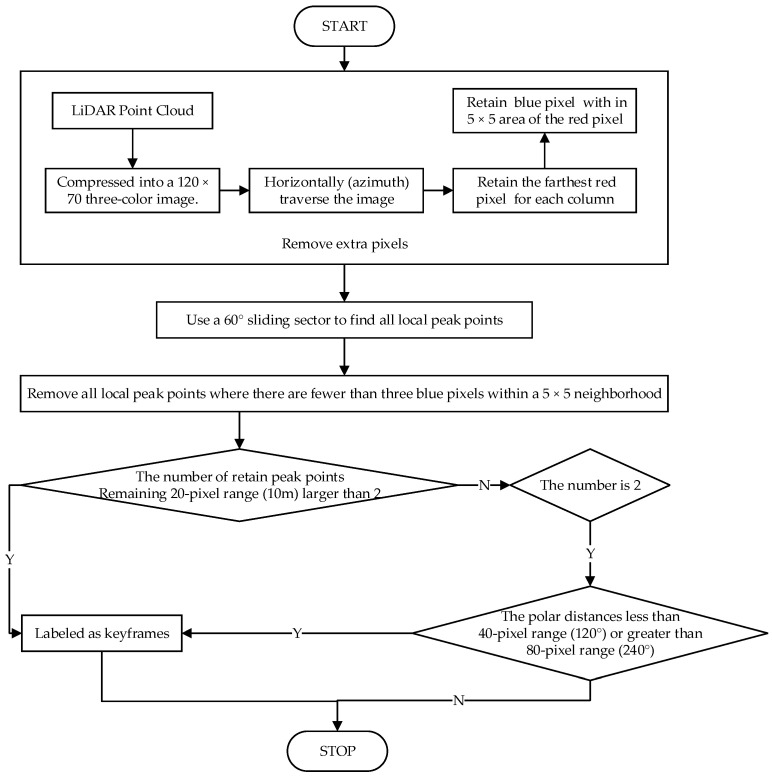
Keyframe Determination Flowchart.

After the above processing, the extracted point cloud is compressed into a 3-channel color image. At this point, further processing is performed. As shown in Figure 9, the steps are as follows:

1. For each angular sector, only retain the farthest ground point and all Obstacle Points within a 5 × 5 area centered on that ground point.

2. Use a 60° sliding sector to traverse and select all local peak points, connecting the beginning and end. Note that these peak points require at least 3 Obstacle Points within a 5 × 5 area centered on them, and their distance must exceed the average distance of the ground points.

3. Mark all point clouds with more than 2 peak points as key frames. At the same time, calculate the polar distances for all point clouds with exactly 2 peak points. Mark all point with polar distances less than 120° or greater than 240° clouds as key frames.

### 2.3. Keyframe Detection Based on MEMS Data

Similar to the classification based on LiDAR point clouds, vehicular motion can also be categorized into the following scenarios:

(a) Vehicle at stationary,
(b) Vehicle in uniform linear motion,
(c) Vehicle undergoing significant and continuous acceleration or deceleration, and
(d) Vehicle undergoing significant and continuous unidirectional angular change.

Evidently, scenario (b), similar to scenario (d) in the LiDAR point clouds, represents the vehicle in a state of regular driving. In this state, the surrounding environment typically exhibits common features, high repetitiveness, and is difficult to describe solely using MEMS measurement data. Conversely, scenarios (a), (c) and (d) correspond to the maneuvering states where the vehicle deviates from typical driving conditions. These maneuvering states generally indicate a higher probability of encountering surrounding environments with distinctive and representative features. In other words, the point clouds acquired in these scenarios can be extracted as keyframes. While ASL-SLAM introduces a notable scenario involving a vehicle passing over a speed bump, this scenario is less relevant to the proposed study due to the infrequent presence of speed bumps on major urban roads. Therefore, the discussion will primarily focus on scenarios (a), (c), and (d) as the target detection cases, assuming the absence of similar situations.

Due to the constraints of vehicle kinematics, this paper assumes that under normal circumstances, the motion of a vehicle should be confined within the following framework [20,21]:

(1) The vehicle can be approximated as a rigid body.
(2) In the absence of turning maneuvers, the vehicle’s motion within its body frame should primarily consist of forward/backward velocity.
(3) The vehicular roll and pitch angles typically do not exhibit significant changes.
(4) The vehicular vertical velocity remains relatively stable.

Under these assumptions, the sensor states corresponding to the three scenarios (a), (c), and (d) can be simplified as follows:

Scenarios (a): The front and right accelerometers, as well as the yaw rate gyroscope, exhibit near-zero readings.

Scenarios (c): The forward accelerometer shows significant changes, while the lateral accelerometer and gyroscope exhibit minimal variations.

Scenarios (d): The yaw rate gyroscope shows continuous changes, the sign of the lateral accelerometer reading is opposite to that of the yaw rate, and the forward accelerometer reading is negative or close to zero.

**Figure 10:**
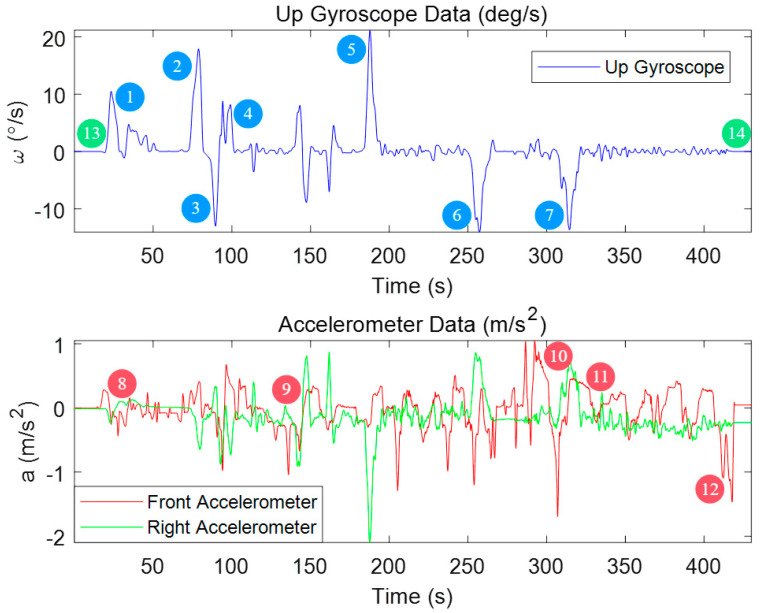
MEMS Gyroscope and Accelerometer Data Variations During Experiment.

Figure 10 illustrates the variations in gyroscope and accelerometer readings during an experiment. As indicated by the color-coded annotations, the red square boxes highlight periods where the vehicle is stationary, the black rectangular boxes represent instances of turning maneuvers, and the blue rectangular boxes correspond to sudden changes in velocity.

Analyzing these motion patterns reveals that the vehicle experiences at least 14 distinct periods suitable for keyframe recording during the experiment, including 7 turns (marked as blue), 5 rapid velocity changes (marked as red) and 2 stationary phases (marked as black). The periods corresponding to scenarios (a), (c) and (d) are classified as strong keyframe intervals.

### 2.4. Keyframe Recording

The keyframe recording and processing are similar to Section 2.2.3, where the point cloud is divided into several bins based on azimuth and distance, and their characteristics are recorded for subsequent loop-closure comparison. The difference here is that an angular interval of 6° and a distance interval of 1 m are used, with an upper limit of 80 m, thereby compressing the point cloud into a 60 × 80 image.

**Figure 11:**
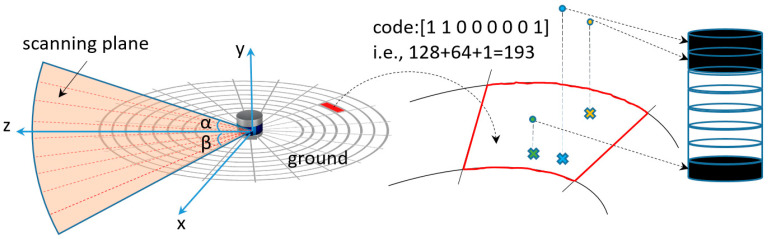
Improved SCAN CONTEXT Point Cloud Compression.

As shown in Figure 11, unlike the traditional SCAN CONTEXT method, which only extracts the maximum height values of the bins as the representative characteristics, the point cloud is divided into 8 height regions from −2 m to 22 m, with each region being 3 m apart. Then, if there are points within the specified region, a ‘1’ is used for identification; if there are no points, a ‘0’ is recorded. Under this encoding scheme, the point cloud information can be recorded as grayscale data ranging from [0, 255]. 

**Figure 12:**
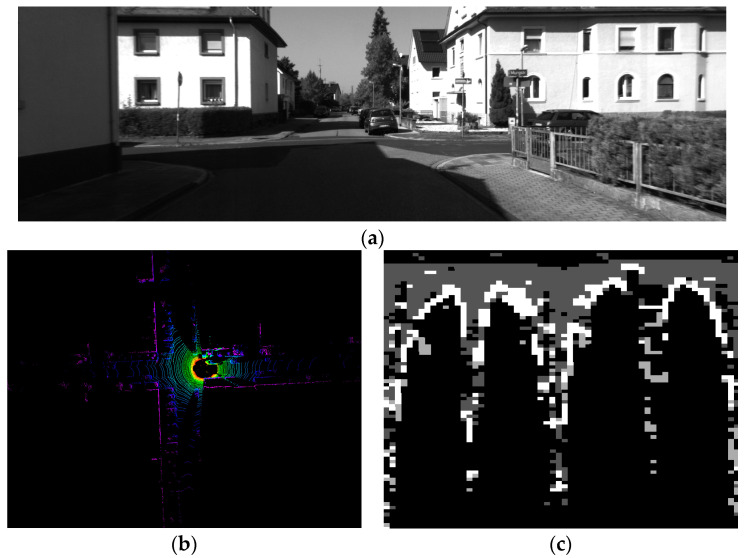
Camera Images, LiDAR Point Cloud and Re-Encoding Result of the Cross Road. (**a**) View of Crossroad from Camera. (**b**) LiDAR Point Cloud of Cross. (**c**) Encoding Result of LiDAR Point Cloud.

Figure 12 depicts the LiDAR point cloud and the grayscale image after re-encoding with the proposed method. In the subsequent loop closure detection of the point cloud, the similarity is checked at first, and the loop closure is confirmed only when the similarity between the two images reaches a certain threshold. In this paper, the method for detecting similarity follows the approach proposed in SCAN CONTEXT [14], which involves calculating the cosine distance between two candidate frames column-wise and averaging the results across all columns. The outcome is a value between 0 and 1, where values closer to 0 indicate higher similarity. We have chosen a similarity threshold of 0.4, which is relatively relaxed. This threshold serves as an initial filter to identify potentially similar frames. Once two frames are preliminarily identified as similar, ICP is employed to compute the translation and rotation matrices between them [11]. Only when the translation distance is below 4 m and the roll and pitch angles are less than 10° is loop closure between both point clouds definitively confirmed.

In contrast to the LiDAR iris for a loop [22], this paper significantly reduces the number of candidate loop closure frames. For the subsequent rapid matching and further refinement of loop closure frames, the algorithm proposed in this study adopts the following approach:

(a) Compressed information is stored in two formats: one as a standard grayscale image, and the other as a binary image (where any non-zero point is marked as 1).

(b) The binary image is utilized for rapid inter-frame matching. Subsequently, for keyframes that achieve a certain threshold of inter-frame matching rate, further matching is performed using the grayscale images.

## 3. MEMS-LiDAR Integrated Navigation System

**Figure 13:**
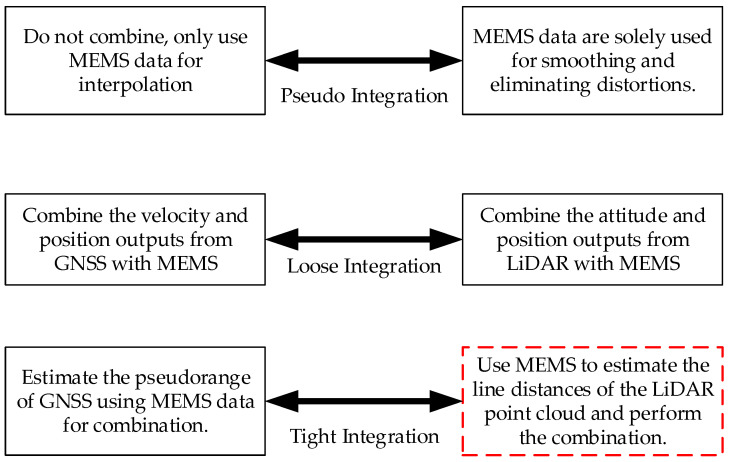
Use of Traditional MEMS/GNSS-Integrated Navigation Systems to Redefine LiDAR-Integrated Navigation Systems.

**Figure 14:**
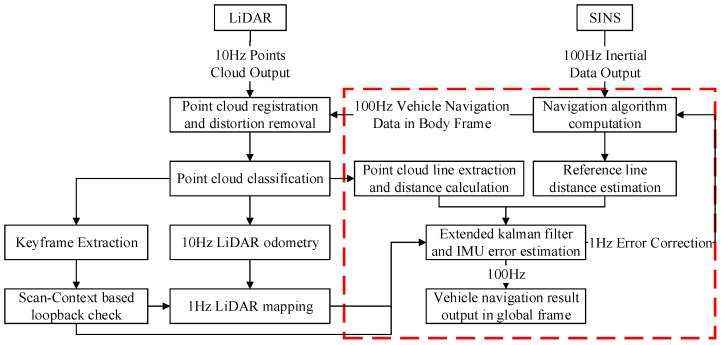
Algorithm Overview of SINS-Based 3D LiDAR Tightly Integrated with SLAM.

As shown in Figure 13, the combination methods of LiDAR and MEMS can be categorized from simple to complex in the form of pseudo integration [14], loose integration [23], and tight integration [24]. Among them, tight integration utilizes the fusion of line features extracted from LiDAR data with MEMS navigation data. The core of the integrated algorithm is depicted in Figure 14, which focuses on the extraction and application of line distances [24]. This integrated approach primarily offers the advantage of reducing the demand for the structural complexity of the environment to a certain extent.

## 4. Experiment

### 4.1. Comparison Results Based on Public Datasets

**Figure 15:**
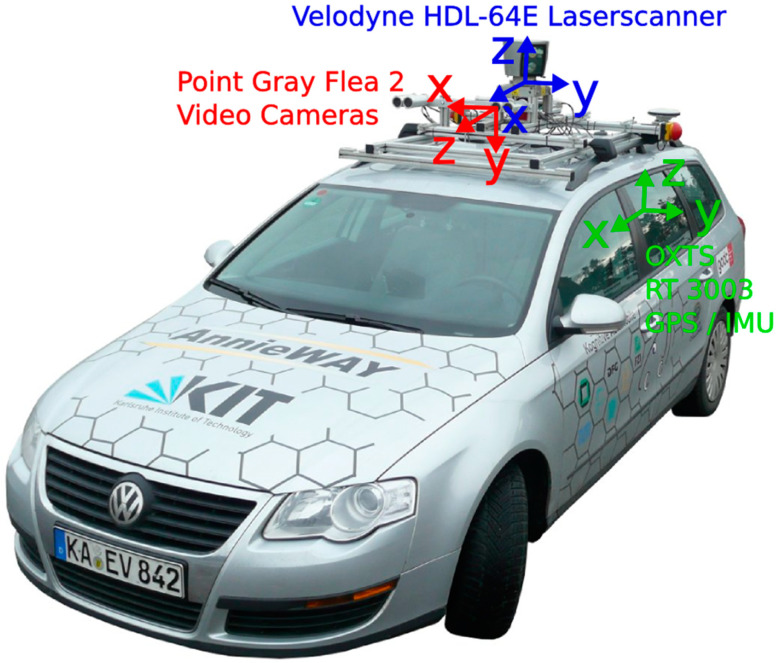
The sensor configuration on the platform.

The KITTI Vision Benchmark Suite [25] is a renowned dataset widely used in computer vision and robotics research, particularly for tasks related to autonomous driving. Captured using a sensor suite mounted on a moving vehicle, it comprises a rich variety of real-world driving scenarios across different environments, weather conditions, and traffic situations. As shown in Figure 15 [26], the dataset provides synchronized data from various sensors, including high-resolution color and grayscale cameras, a Velodyne LiDAR scanner, and a precise GPS/IMU localization system. KITTI’s diverse modalities and accurate ground-truth annotations for tasks like object detection, tracking, semantic segmentation, optical flow, and visual odometry have made it an invaluable resource for developing and evaluating algorithms in applications like self-driving cars, mobile robotics, and 3D scene understanding.

**Figure 16:**
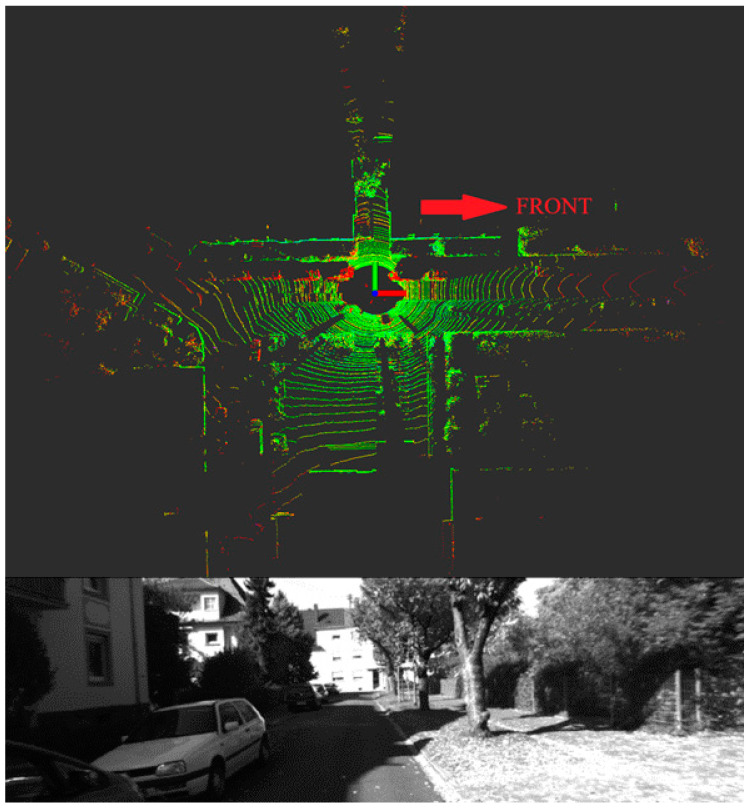
Camera Image and Point Clouds from KITTI Dataset 00.

Dataset 00, the most frequently utilized and benchmarked sequence in the KITTI dataset, captures a wide array of urban driving scenarios, such as straight stretches, roundabouts, and traffic congestion. Figure 16 illustrates the LiDAR point cloud and the corresponding left camera image for a frame extracted from this dataset. This comprehensive dataset serves as an excellent benchmark for assessing the generalizability of the proposed algorithm across various driving conditions.

**Figure 17:**
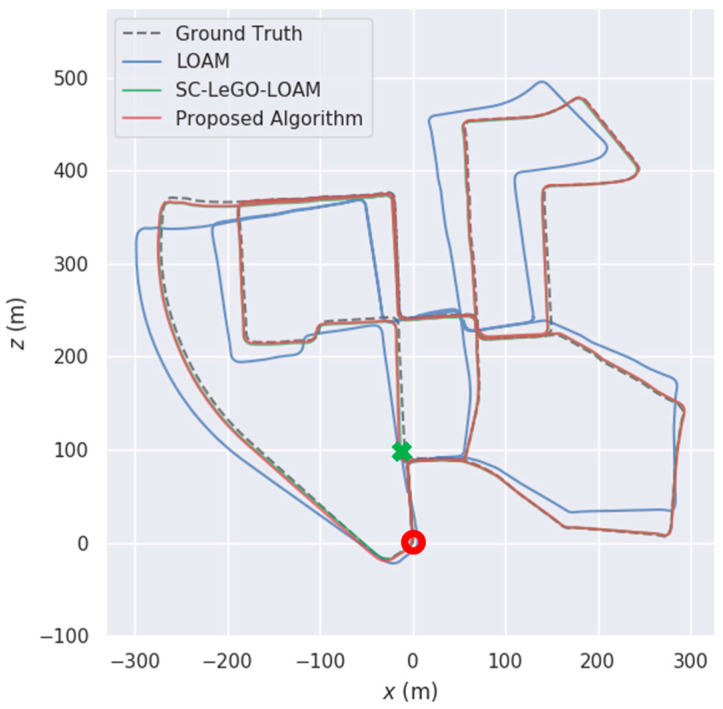
Comparison Results of KITTI Dataset 00.

As illustrated in Figure 17, the vehicle starts from the red circle point and ultimately returns to the green cross section, having traveled a total of 3724.19 m and experienced 8 loops. A comprehensive comparison with other state-of-the-art methods showcases the performance of the proposed algorithm. It achieves near-identical accuracy to SC-LeGO-LOAM, significantly outperforming methods with no loop closure strategies, such as LOAM. Furthermore, across all 8 loop closure opportunities present in this dataset, the proposed algorithm achieves a 100% identification rate. This highlights its ability to reduce computational burden associated with loop closure without compromising accuracy. 

Table 1 presents a detailed performances comparison of different algorithms on the KITTI dataset 00. The results indicate that the proposed method demonstrates enhanced robustness in typical driving scenarios. Specifically, it achieves a 6% improvement in maximum error and a 7% improvement in overall error compared to SC-LeGO-LOAM, which is the highest performing baseline method. These demonstrations highlight the effectiveness of the proposed approach in achieving higher accuracy in common driving conditions.

Starting from Section 4.2, the paper presents the experimental results of the proposed algorithm on a local dataset. Unlike the KITTI dataset, which only contains LiDAR data, the local dataset includes both LiDAR and MEMS data, allowing for a more comprehensive evaluation of the algorithm’s efficiency in keyframe extraction for loop closure.

### 4.2. Sensors System

All the sensors are mounted on a Sport Utility Vehicle (SUV) for data collection. The LiDAR unit and the GNSS antennas installed on the roof, while the SINS equipment and power supply are secured within the SUV. 

**Figure 18:**
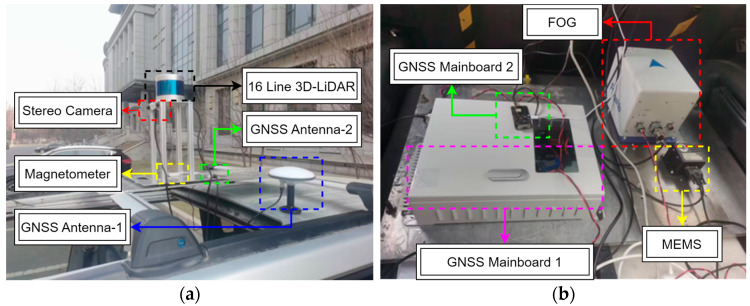
Experimental Hardware. (**a**) 3D LiDAR and GNSS Antenna. (**b**) GNSS Processing, Fiber Optic Gyroscope, and MEMS.

Figure 18 depicts the experimental setup, which utilizes a high-performance fiber optic gyroscope navigation system integrated with a GNSS receiver to provide ground truth data. Table 2 summarizes the specifications of this system. Precise alignment of sensor positions is crucial for minimizing navigation errors arising from lever arm effects. Therefore, the central positions of all sensors are carefully measured and aligned with the azimuth axis. Detailed measurement data are available in Table A1. Note that this table omits sensors with less stringent relative positioning requirements, such as the magnetometer used for initial SLAM orientation.

The LiDAR sensor employed in this study is the Leishen 16 Line 3D-LiDAR, and the performance parameters are detailed in Table 3.

Table 4 lists the performance parameters of the ADIS16445 MEMS-IMU, a complete inertial system comprising a tri-axial gyroscope and a tri-axial accelerometer. The UM6 magnetometer provides a static heading with an accuracy of better than 2°, serving as the initial heading for the system. Similarly, the GNSS receiver provides the initial longitude, latitude, and altitude.

Data processing is performed on a laptop equipped with an Intel i7-6700 CPU, a GT960m graphics card, and 12GB of RAM. The operating system is Ubuntu 16.04, running the ROS (Robot Operating System) kinetic distribution. This software environment supports both sophisticated data simulation and advanced graphical rendering.

### 4.3. Experimental Area

All data in the paper are collected in April 2023 at Harbin Engineering University and its surrounding areas, with geographic coordinates approximately at 126.68° Longitude and 45.77° Latitude, and an elevation of about 130 m. Based on the actual driving environment, the driving speeds of the SUV in different experiments are controlled between 15 km/h and 30 km/h.

### 4.4. Result and Analysis

LOAM and SC-LeGO-LOAM are two commonly used comparative algorithms among non-machine learning-based LiDAR SLAM algorithms [24s] [26]. The former is designed as a loop closure-free algorithm, while the loop closure algorithm proposed in this paper is based on an improvement of the latter. Therefore, these two algorithms are chosen for comparison in the experimental results. In addition, most algorithms other than these two are derived algorithms, enhancing the applicability of the algorithms in certain specific scenarios. Consequently, this paper does not conduct a specific comparison with them.

**Figure 19:**
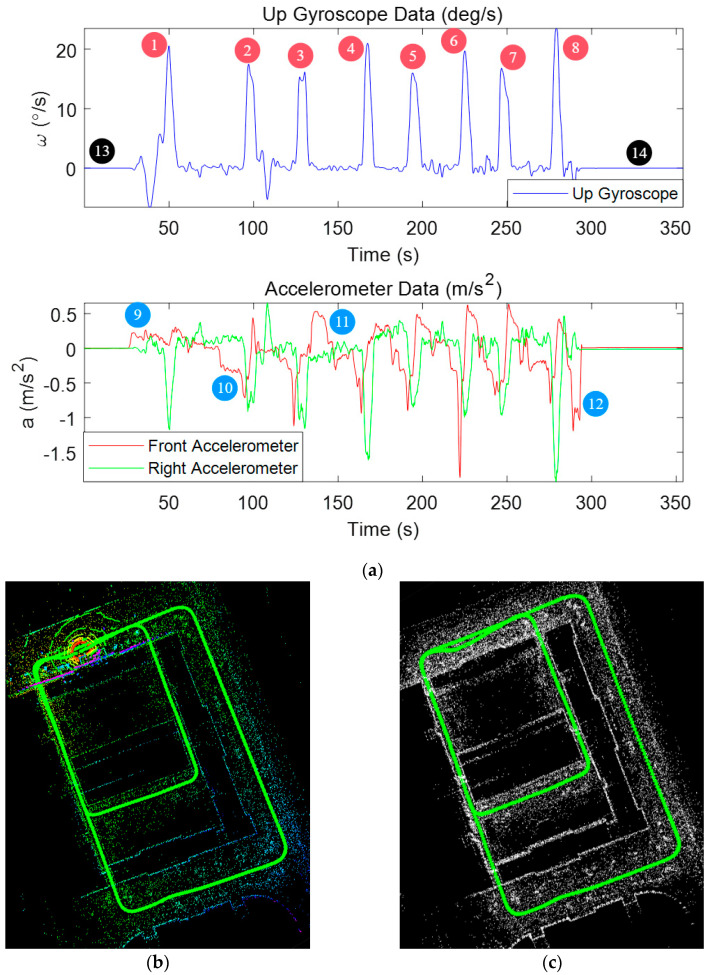

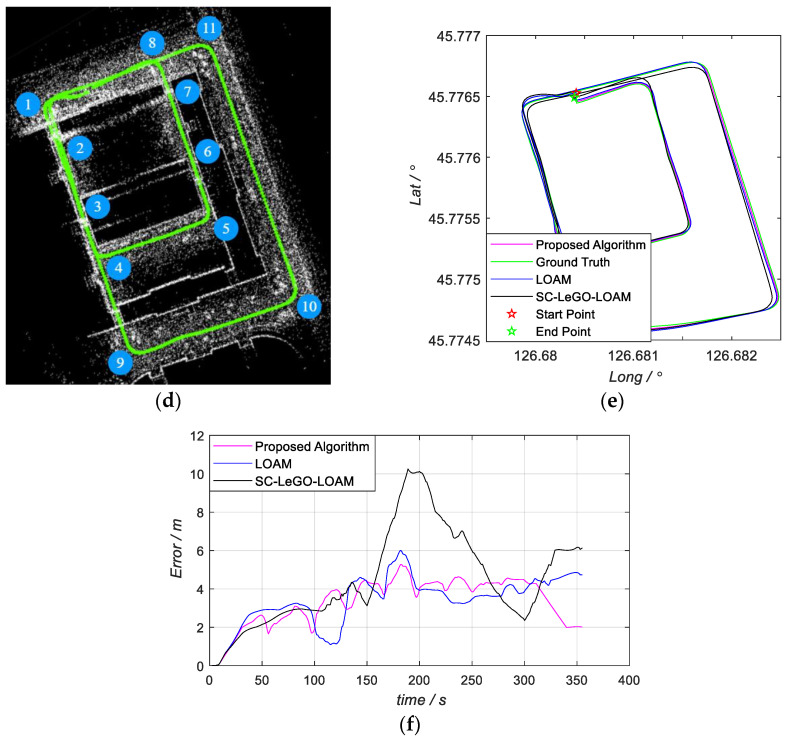
Result of LiDAR SLAM in Data_1. (**a**) MEMS data from Data_1. (**b**) Built by LOAM. (**c**) Built by SC-LeGO-LOAM. (**d**) Mapping Result of Method from Paper. (**e**) Direct Comparison of Various Algorithms. (**f**) Positioning Errors of Algorithms Measured in Meters.

Data_1 was collected at 6 PM on 4 April 2023, near Building 61 of Harbin Engineering University, with a total travel distance of 1460 m. In this scenario, the SUV’s circles around the building for two laps, and the main purpose of the scene setup is to verify the effectiveness of the algorithm in the paper under general environmental conditions. Table 5 presents a performance comparison of different SLAM algorithms under this set of data. The trajectory map can be seen in Figure 19, while the MEMS data in Figure 19a show that it has 14 potential loop closure locations. The LiDAR point cloud in Figure 19d records 11 potential loop closure locations. In the actual experimental process, LiDAR loop closure points 1, 2, 3, 4, 5, 6, 7, and 8 complete actual loop closures, and MEMS loop closure points 13 and 14, as well as 1 and 5 (corresponding to LiDAR loop closure location 1), achieve actual loop closures. Ignoring the repeated paths, the actual loop closure locations in this experiment total 3, corresponding to LiDAR loop closure points 1, 2, and 5, and achieving a 100% recall rate in the experiment. In the initial 1000 m, SC-LeGO-LOAM maintains relatively good performance. However, after the final incorrect loop closure, the total distance is re-optimized, leading to significant misalignment between the final distance and the azimuth angle. The method presented in this paper performs similarly to LOAM in the early stages, but because the original LOAM lacks loop closure detection functionality, its errors are bound to increase over time.

**Figure 20:**
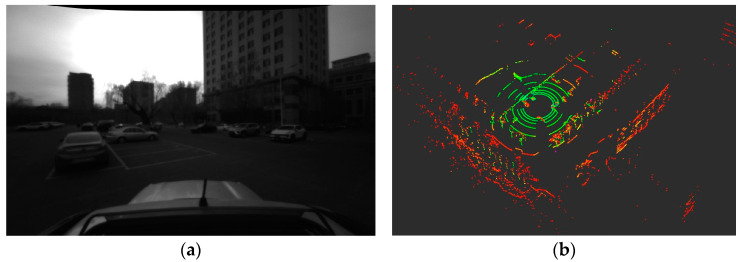
Camera Image and Point Clouds Near Parking Lot. (**a**) Camera Image. (**b**) Point Clouds.

**Figure 21:**
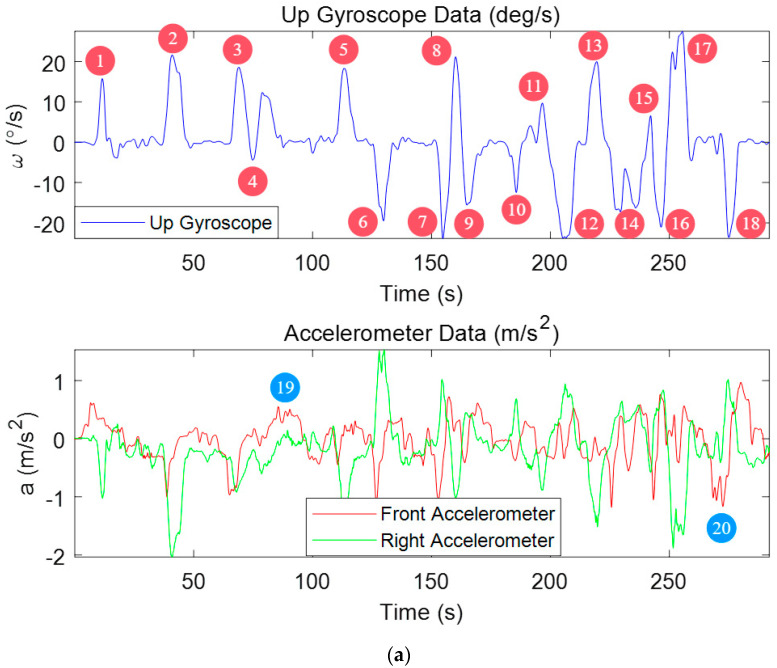

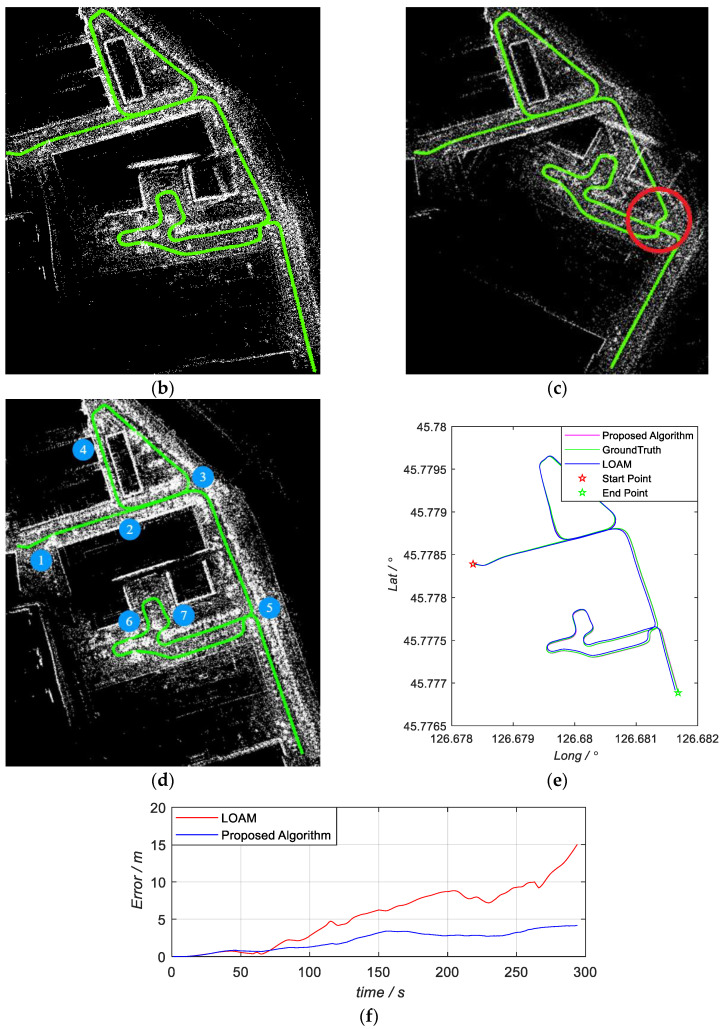
Result of LiDAR SLAM in Data_2. (**a**) MEMS Data from Data_2. (**b**) Built by LOAM. (**c**) Built by SC-LeGO-LOAM. (**d**) Mapping Result of Method from Paper. (**e**) Direct Comparison of Various Algorithms. (**f**) Positioning Errors of Algorithms Measured in Meters.

Data_2 was collected at 5 PM on 5 April 2023, near the parking lot of Harbin Engineering University, with a total travel distance of 1403 m. As shown in Figure 20, the structural feature weakens near the parking lot, and the lack of structural features causes matching issues with the algorithm that rely on points. The experiment is mainly designed to demonstrate the stability of the algorithm in the paper relative to the comparative algorithms under the present conditions of the paper. Table 6 presents a performance comparison of different SLAM algorithms under this set of data. The trajectory of the experimental route is visualized in Figure 21. Figure 21a highlights 20 potential loop closure locations based on the trajectory, while Figure 21d shows the LiDAR point cloud map with 7 potential loop closure locations. During the experiment, loop closures are successfully achieved at locations 2, 3, and 5, as identified from the LiDAR data. Additionally, loop closures are achieved at locations 2 and 6 (corresponding to LiDAR location 2), and 7 and 18 (corresponding to LiDAR location 5), as identified from the MEMS data. Considering only unique loop closure locations, this experiment involves 3 actual loop closure points, corresponding to LiDAR locations 2, 3, and 5. Our proposed method achieves a 100% recall rate, successfully identifying and closing all actual loop closures. Judging from the comparison of the results, the incorrect loop closure (red circle) by SC-LeGO-LOAM leads to severe issues once again, causing the method to fail entirely in this set of experiments. After the first loop closure, LOAM begins to accumulate heading errors, resulting in the continuous amplification of positioning errors in the subsequent SLAM due to the heading deviation.

The remaining data were also collected from 3 to 5 April 2023, at Harbin Engineering University and its surrounding areas. Data_3 has a loop closure point available shortly after the start, which can be used to eliminate accumulated errors. Data_4 features a longer segment of nearly straight-line travel. These two sets of experiments are not designed intentionally with specific scenarios; they are standard test experiments. Hence, they are not elaborately compared in detail within the text, but merely listed in Table 7 where a comparison of the positioning errors of different algorithms is presented.

## 5. Discussion

Considering the limited computational resources available on vehicle platforms, this paper focuses on enhancing traditional LiDAR SLAM algorithms rather than adopting computationally demanding deep learning approaches. While deep learning has shown impressive results in LiDAR SLAM, its high computational requirements and dependence on powerful hardware restrict its applicability in real-time autonomous driving systems [27,28].

The proposed scenario-based LiDAR keyframe extraction algorithm, coupled with the modified point cloud compression mechanism, demonstrates significant performance improvements in both the KITTI-00 dataset and the local dataset. This approach effectively identifies loop closure points while minimizing false positives, especially in environments with sparse structural features. These results indicate that substantial performance gains can be achieved even without relying on deep learning by leveraging a deeper understanding of environmental characteristics and vehicle motion information within traditional LiDAR SLAM frameworks.

Importantly, the proposed algorithm effectively reduces the number of candidate frames in loop closure detection without missing any true loop closure points. This is achieved by carefully selecting keyframes based on their suitability for loop closure, informed by both LiDAR and MEMS data. Furthermore, by utilizing context information that better represents the structural characteristics of the point cloud to record candidate frames, the algorithm achieves a lower mismatch rate in subsequent loop closure detection processes. This advantage is expected to become more pronounced as the navigation distance increases, leading to a more efficient and accurate loop closure process.

However, it is important to note that all experiments in this paper are conducted over relatively short distances and under the assumption of complete GNSS signal unavailability. This differs from real-world autonomous driving scenarios, which often involve longer distances and more complex environments. More importantly, while urban environments may experience GNSS signal obstruction or interference, GNSS signals are not always in a state of complete loss. Therefore, in future long-distance navigation experiments, we must consider how to effectively utilize intermittently available GNSS information and integrate it with the LiDAR SLAM algorithm proposed in this paper to further enhance overall navigation and localization performance. This combination will not only overcome the limitations of a single technology but also achieve more stable and accurate positioning and navigation in complex and dynamic urban environments.

## 6. Conclusions

This paper addresses the crucial question of “when-to-loop” by proposing a novel keyframe extraction algorithm for LiDAR loop closure. The key contributions and findings of this study are as follows:

1. Development of a new keyframe extraction method: The proposed approach leverages both LiDAR point cloud characteristics and MEMS data, incorporating vehicle kinematic constraints to identify optimal loop closure opportunities.
2. Enhanced point cloud processing: For LiDAR-based keyframe extraction, multiple segmentations are performed on the point cloud, taking vehicle height into account. This segmented data is then compressed into a three-color image used for keyframe determination, significantly reducing the number of keyframes for loop closure matching.
3. Utilization of MEMS data: Three specific scenarios are introduced by utilizing MEMS data in loop closure: stationary phases, acceleration/deceleration periods, and cornering maneuvers. A secondary keyframe extraction is performed by analyzing MEMS data patterns within these scenarios and incorporating vehicle kinematics, further refining loop closure candidates.
4. Improved loop closure detection: The proposed algorithm departs from the maximum height approach used in SC-LeGO-LOAM, instead employing a structural framework for point cloud compression. This leads to successful identification of all loop closure points while significantly reducing false positives.
5. Significant performance improvements: Experimental results demonstrate an overall improvement in accuracy of 17% for the proposed local dataset compared to traditional methods.

This study not only achieves significant algorithmic improvements over existing methods but also paves a new technological pathway for autonomous driving and robotic navigation applications.

## Figures and Tables

**Table 1:** Performances of SLAM Methods for KITTI Dataset 00.

Method	LOAM	SC-LeGO-LOAM	Proposed Algorithm
Avg. Positioning Error (m)	3.76	2.76	2.81
Max Positioning Error (m)	31.13	11.75	10.83
Travel Distance Error (m)	13.2	7.7	7.3

**Table 2:** Specifications of Reference Integrated Navigation System.

Reference Accuracy	Specifications
Pitch	<0.02°
Yaw	<0.02°
Heading	<0.05°
Velocity (Integrated)	<0.1 m/s
Positioning (Integrated)	<1 m
Output Rate	100 Hz

**Table 3:** Performance Parameters of 3D-LiDAR.

Performance	Parameters
Detection Range	200 m
Point Rate	320,000 pts/s (single echo)
Distance Measurement Accuracy	±3 cm
Laser Wavelength	905 nm
Maximum Echo Count for Reception	2
Scanning Channels	16
Field of View Angle	360° × −15°~15°
Scanning Frequency	5~20 Hz
Angular Resolution	0.09°@5 Hz
Power Supply Range	9~36V DC
Operating Temperature	−20 °C~55 °C

**Table 4:** Performance Parameters of ADIS16445.

Performance	Parameters
Gyroscope Dynamic Range	±250°/s
Gyroscope Sensitivity	0.01°/s
Gyroscope Nonlinearity	±0.1%
Gyroscope Bias Stability	12°/h
Angular Random Walk	$0.56{}^{\circ}/\sqrt{h}$
Accelerometer Dynamic Range	±5 g
Accelerometer Sensitivity	0.25 mg
Accelerometer Nonlinearity	±0.2%
Accelerometer Bias Stability	0.075 mg
Velocity Random Walk	$0.0735\;m/s/\sqrt{h}$
Bandwidth	330 Hz
Output Rate	100 Hz

**Table 5:** Performance of SLAM Methods for Data_1.

Method	LOAM	SC-LeGO-LOAM	Proposed Algorithm
Avg. Positioning Error (m)	3.89	4.14	3.33
Final Heading Error (°)	2.17	5.43	2.32
Max Positioning Error (m)	6.17	10.12	5.32
Travel Distance Error (m)	17	18	13

**Table 6:** Performance of SLAM Methods for Data_2.

Method	LOAM	SC-LeGO-LOAM	Proposed Algorithm
Avg Positioning Error (m)	5.79	Failed	2.25
Final Heading Error (°)	6.22	Failed	1.02
Max Positioning Error (m)	15.17	Failed	4.12
Travel Distance Error (m)	22	Failed	11

**Table 7:** Comparison of Positioning Errors for Data_3 and Data_4.

Data	LOAM	SC-LeGO-LOAM	Proposed Algorithm	Travel Distance
Pos error of Data_3 (m)	28.79	18.12	14.17	2205
Pos error of Data_4 (m)	16.73	9.25	10.34	2234

## Data Availability

The datasets presented in this article are not readily available because they are part of an ongoing study. Requests to access the datasets should be directed to the corresponding author.

## Appendix A

**Table A1:** Positions of Different Sensors Relative to FOG Center.

Sensors	Right	Forward	Up
MEMS	5.3 cm	−22.5 cm	−4.3 cm
LiDAR	−32.5 cm	78 cm	113.5 cm
GNSS Antenna	21.5 cm	68.5 cm	83 cm
